# Letter from the Editor in Chief

**DOI:** 10.19102/icrm.2019.101208

**Published:** 2019-12-15

**Authors:** Moussa Mansour


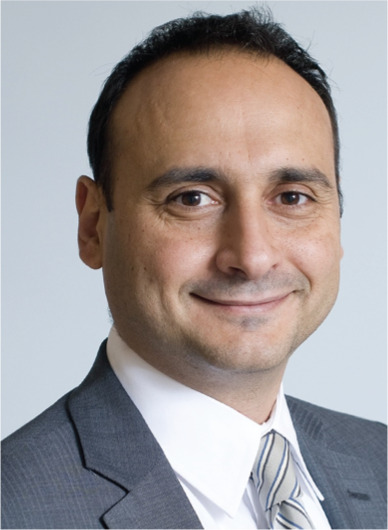


Dear Readers,

The management of patients undergoing the extraction of cardiac implantable electronic devices (CIEDs) can demonstrate varying degrees of difficulty depending on these individuals’ unique anatomical and disease characteristics. Among patients who are dependent on pacing, a temporary solution as a bridge to reimplantation of the permanent device is necessary and, often, an externalized permanent device is used. However, this strategy can be associated with prolonged hospitalization and may be uncomfortable for the patient. Related to this topic, Gonzales et al. offer an interesting article in this issue of *The Journal of Innovations in Cardiac Rhythm Management* titled “Comparison of Leadless Pacing and Temporary Externalized Pacing Following Cardiac Implanted Device Extraction.”^1^ Here, they compare the outcomes of using leadless pacing and externalized devices, respectively, in the immediate postextraction period. While the study is retrospective and included a small number of patients, it is nonetheless important because the proposed strategy may facilitate improved care of patients after device extraction.

Leadless pacing was introduced a few years ago and its use has been expanding. It provides many advantages over conventional pacing, including the avoidance of long-term complications associated with intravascular leads. Moreover, because leadless devices contain less hardware, they may be less likely than conventional pacemakers to lead to infection. At this time, leadless pacing can only be performed in the right ventricle. However, studies are ongoing to test its use in left ventricular pacing^2^ and also in combination with subcutaneous defibrillation.^3^ Once commercially available, these technologies are expected to provoke a rapid expansion of leadless pacing, potentially leading it to replace conventional pacing and defibrillation entirely. As described in the article mentioned above, postextraction patients will particularly benefit from such future technologies.

I hope that you enjoy reading this issue of *The Journal of Innovations in Cardiac Rhythm Management*. I also would like to wish you happy holidays and a prosperous and healthy 2020!

Sincerely,


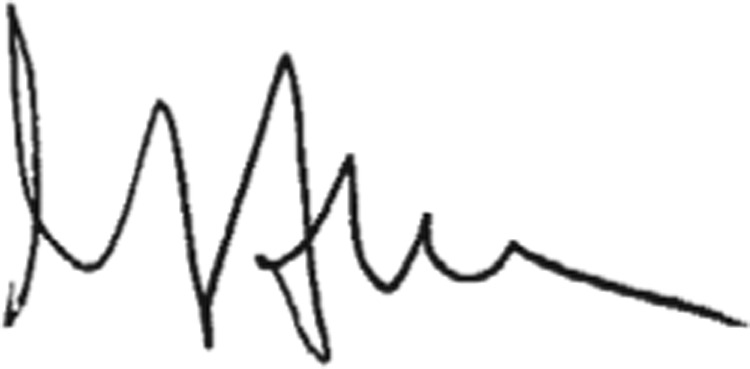


Moussa Mansour, md, fhrs, facc

Editor in Chief

The Journal of Innovations in Cardiac Rhythm Management

MMansour@InnovationsInCRM.com

Director, Atrial Fibrillation Program

Jeremy Ruskin and Dan Starks Endowed Chair in Cardiology

Massachusetts General Hospital

Boston, MA 02114
